# 4.5: Diagnosing breast cancer in a high-risk cohort

**DOI:** 10.1186/bcr3501

**Published:** 2013-11-08

**Authors:** EAM O'Flynn, V Houle, ARM Wilson, AZ Evans, SD Allen

**Affiliations:** 1 Institute of Cancer Research and Royal Marsden Hospital, Sutton, UK; 2 Royal Marsden Hospital, London, UK; 3 Ninewells Hospital and Medical School, Dundee, UK

## Introduction

MRI is a common method for detecting breast cancer in women at high risk [1,2] These women may instead be diagnosed mammographically or present symptomatically. The aim of this study was to investigate how breast cancer is diagnosed in high-risk women and determine whether there are specific characteristics related to the type of presentation.

## Methods

A total of 125 high-risk patients with 134 breast cancers (69 *BRCA*, 65 family history) were managed at the Royal Marsden Hospital from 1994 to 2013. Following ethical approval, data were collected retrospectively for each presentation of breast cancer: method of presentation/diagnosis (MRI, mammography, symptomatic), age at diagnosis, cancer type, grade, size, presence of DCIS, lymphovascular invasion (LVI), nodal status and tumour subtype. Chi-squared and ANOVA analyses determined any association between the parameters, *P *< 0.05 was significant.

## Results

Ten breast cancers were MRI detected, 43 mammography detected and 81 symptomatic (mean age 41, 51, and 45 years (*P *= 0.008); mean size 17, 29, and 34 mm (*P *= 0.076) respectively).The majority of cancers were high-grade (68%) invasive ductal carcinomas (78%) without LVI (76%). MRI-detected cancers were triple negative in 60% (*P *= 0.03), node negative in 100% (*P *= 0.005) with DCIS in 70% (*P *= 0.007). Mammography-detected cancers were luminal in 77% (*P *= 0.03), node negative in 77% (*P *= 0.005), with DCIS in 81% (*P *= 0.007). Symptomatic cancers were luminal in 54%, triple negative in 41%, node negative in 56% and DCIS positive in 51%.

## Conclusion

In this high-risk cohort, MRI detects small, triple-negative, node-negative cancers in younger women, while mammography detects larger, luminal, cancers in older women that may be node positive.
